# Validation of the “Mind the Gap” Scale to Assess Satisfaction with Health Care among Adolescents

**DOI:** 10.4274/balkanmedj.galenos.2018.2017.0168

**Published:** 2019-02-28

**Authors:** Evrim Kızıler, Dilek Yıldız, Berna Eren Fidancı

**Affiliations:** 1 Department of Pediatric Nursing, Ankara Yıldırım Beyazıt University Faculty of Health Sciences, Ankara, Turkey; 2 Department of Pediatric Nursing, Health Sciences University Faculty of Nursing, Ankara, Turkey

**Keywords:** Adolescent, diabetes mellitus, Mind the Gap scale, patient satisfaction, transitional care, validity and reliability

## Abstract

**Background:**

At present, more than 90% of adolescents with chronic conditions survive into adulthood as health care users and move pediatric to adult care with their chronic illness. Therefore, the need satisfaction scale focuses specifically on transitional care and reflect the increasing expectations among youth and their parents.

**Aims:**

To examine the validity and reliability of the Turkish version of Mind the Gap scale.

**Study Design:**

Methodological study.

**Methods:**

The Turkish versions of Mind the Gap scale and Patient Assessment of Choronic Illness Care scale were applied to the participants in two tertiary hospitals in Ankara. The validity was evaluated with factor analyses and content-scope validity; the reliability was evaluated with item-total score correlation, internal consistency, and continuity methods.

**Results:**

A total of 109 adolescents and 157 parents completed the questionaire. The content validity was confirmed. Exploratory factor analysis was used to determine the factor structure of the scale. Both adolescent and parent scales formed three sub-dimensions and explained 71% and 73% of the variation, respectively. The Cronbach’s alpha reliability coefficient of Mind the Gap scale 1 and Mind the Gap scale 2 were 0.89 and 0.87, respectively, with internal consistencies of the parent’s scales reaching 0.92 and 0.90. The test-retest reliability coefficients totalled 0.88 and 0.85 for the adolescents and parents, respectively. The suitability of the model was examined with confirmatory factor analysis. Conformity indices and x2/df value of the model were in good fit to data.

**Conclusion:**

The Turkish version of the Mind the Gap scale is a valid and reliable scale for evaluating the needs, expectations, and satisfaction of adolescents and their parents in terms of health care.

The life expectancy of children with chronic conditions has risen over the past few years. Today, most adolescents with chronic diseases transition to adulthood (1). The successful transition interventions for chronically ill youth from pediatric to adult care also gained importance. The American Academy of Pediatrics emphasizes the importance of high-quality, age-appropriate, and uninterrupted health care services as a person transitions from adolescence to adulthood and providing self-management and independent living activities to adolescents (2,3,4,5,6). This purposeful and high-quality health care transition process, which starts in the early adolescence, aims to maximize the lifelong functioning and well-being of youth with special healthcare needs (2,7).

The quality of health care is assessed by the care satisfaction of the patients. Studies evaluating care satisfaction are commonly performed in the adult population (8). These studies show that the care satisfaction in adults affects the adjustment to care procedure, symptom management, continuity of care, trusting the healthcare providers, and decrease in hospital admissions (9,10,11,12,13). However, studies evaluating care satisfaction in children and adolescents are quite limited and these studies focus on evaluating expectation and needs of children and adolescents rather than evaluating care satisfaction (7,8,14,15). The existing patient satisfaction surveys evaluate the services from the care provider’s point of view, neglect the user’s expectations. In our country, no satisfaction scale focuses specifically on transitional care nor reflect the youth and their parents’s expectations and needs. However, the care quality and patient satisfaction must be evaluated from the patient’s perspective to provide effective communication with individuals with chronic conditions and include them in the treatment process (8,16).

This study aimed to evaluate (i) the validity and reliability of the Turkish “Mind the Gap scale” (MGS) to evaluate the transition health services satisfaction in adolescents with diabetes and their parents. The scale, which is focused on the transition care, is expected to contribute to the assessment of the needs and satisfaction of adolescents and their parents.

## MATERIALS AND METHODS

### Design and participants

This methodological study was conducted with volunteers and randomly selected adolescents (n=109) and accompanying parents (n=157) who were recruited from two pediatric endocrinology clinics of two tertiary hospitals in Ankara. The inclusion criteria for adolescents were as follows: (i) followed-up diagnosis of diabetes at least one year where the study was conducted; (ii) age between 14-21 years old; (iii) ability to read and understand Turkish. The adolescents were excluded from the study if they presented diabetes-related complications and diabetes-related or unrelated neurological problems as they might alter the perspective of diabetes and diabetes care. A total of 5-10 subjects were recommended for each item to achieve the validity and reliability studies (17).

### Procedure

The data were obtained by using the individual questionnaire based on self-evaluation, Turkish MGS, and Turkish Patient Assessment of Choronic Illness Care. Written informed consent was obtained from all participants. The project was approved by the local ethics committee (ethics committe no: 50687469-1491-164-15/1648-4-289). The data collection period was approximately 30 min per participants. As a re-test, after 3 weeks, the scale was filled by 54 adolescent with diabetes to assess the reliability.

### Measures and data

### *Demographic data form*

The demographic data included questions about the age, sex, date of diagnosis, and being informed about diabetes.

### *Mind the Gap scale*

The MGS, which was developed by Shaw et al. (8), is a seven-point Likert scale which allows the assessment of the health care satisfaction of adolescents with chronic conditions and their parents. The construction of the scale was based on multiple inconsistency theories relating to the gap between individual expectations and perceptions (18). The scale consists of four questionnaires, that evaluates the “best care (MGS_1_)” and “current care (MGS_2_)” from adolescents’ and parents’ perspectives separately. A total of 22 items were selected for adolescents and 27 items for parents to assess the interpersonal relationships, health care process, and care environment (Table 1). The difference between the participant’s rating of the “best” and “current” care in the study shows the quality of the transition care.

### *Patient Assessment of Choronic Illness Care*

The scale, which was developed by Glasgow et al. (19), was validated. Patient Assessment of Choronic Illness Care is a simple tool, which consists of 20 items and 5 subscales, to assess the health care among patients with chronic conditions (19). The respondents were asked to rate the items using a five-point Likert scale anchored by “strongly disagree” at 1 and “strongly agree” at 5. The increase in score from the scale indicates the increasing satisfaction of the patient (20).

### Equivalance of language and content validity

After obtaining the permission to adapt the MGS into Turkish, the scale was independently translated by three language experts and two Turkish researchers. Then, the Turkish version was retranslated into English by two other experts in the English language. The final form of the scale was obtained after the expert opinions of two nursing academicians, a biostatistician, and pediatric endocrinologist experienced in transitional care and research methods.

After the language equivalence was established, the scale was tested on 10 participants who were then excluded from the remainder of the study. After the expert opinions, we determined to use the MGS without making any changes on the scale items.

### Statistical analysis

All analyses were performed using the IBM SPSS Statistics for Windows, Version 21.0. Armonk, NY: IBM Corp. The reliability was tested using Cronbach’s alpha coefficients, item-total subscale correlations, and repeatibility of the scale for the complete scale and for each subscale. The self-care scale was used to determine the criterion validity of the scale. Validity was evaluated using the exploratory factor analysis and confirmatory factor analysis. Principal component analysis and varimax rotation were used for exploring the dimensionality. The items with loadings >0.4 were selected as a factor. The Kaiser–Meyer–Olkin measure and Bartlett’s test of sphericity were used to evaluate the sample’s adequacy. The relational assumptions between subscales were compared with oblimin rotation.

### Ethic

The ethical approval for the study was obtained from Gülhane Military Medical Academy (approval number: 50687469-1491-164-15/1648-4-289) and Ankara Childen and Oncology Hematology Training and Research Hospital (approval number: 13.05.2015/18) local ethic committees.

## RESULTS

The present study was conducted with 266 volunteer participants (109 adolescents with diabetes and 157 accompanying parents), who met the inclusion criteria, to evaluate the validity and the reliability of “MGS”.

### Participants’ characteristics

The mean age of the adolescents was 15.28±1.44 years; 53.2% were boys (n=58). The average age at diagnosis was 10.47 (2.0-16.0) years, and the average duration of disease was 4.8 (1.0-15.0) years. The average age of the parents was 41.9±2.17 years; 66.9% were mothers, and 76.5% reached high school or higher education.

### Validity of MGS

### *Exploratory factor analysis*

First, the sampling adequacy was confirmed with the Kaiser–Meyer–Olkin measurement (adolescent: 0.729, parent: 0.787) and Bartlett’s test of sphericity (p<0.01). The test results confirmed the appropriateness of the sample and the sufficient association between variables to perform factor analysis (21). The factor loads were analyzed with the principal component and orthogonal varimax rotation technique and found to be higher than 0.4 (22,23,24). All items in the adolescent and parental forms presented an Eigenvalue higher than 1 and were considered as factors (23,24). According to the exploratory factor analysis results, the adolescent and parents scales consisted of a three-factor structure which explained 71% and 73% of the variation in adolescent and parental scores, respectively (Table 1).

### *Confirmatory factor analysis*

The suitability of the model structure obtained with exploratory factor analysis was tested with confirmatory factor analysis. The first criterion assessed for model suitability; chi-square degrees of freedom statistics (x^2^/df) yielded values of 3.46 (x^2^=377.807; df=109 p=0.000) and 3.157 (x^2^=252.534; df=80; p=0.000) for MGS_1_ and MGS_2_, respectively. According to the confirmatory factor analysis of the MGS_1_ and MGS_2_ for parents, x^2^/df values reached 3.07 (x^2^=199.55; df=65; p=0.000) and 3.40 (x^2^=309.401; df=91; p=0.000), respectively. Table 2 shows the goodness-of-fit index (GFI) values of the scale model obtained in the study. Our study showed that the x^2^/df values showing an overall model fit were in a desirable range, whereas the GFI and adjusted GFI values showed a good fit.

Although most of the GFIs were in acceptable limits, the values of other indices (comparative fit index, incremental fit index, Tucker-Lewis index, root mean square residual index, root mean square residual) were out of acceptable limits (Table 2). Therefore, the model of the scale was analyzed in terms of modification indices and residuals, and causal relationships between the data and model fit indices were evaluated (25). Modification indices and residuals can invalidate the whole model by affecting the coherence between the data and the model or the causal relationships among data (25). None of the variables were excluded from the model given the high values of modification indices that indicate the relationship between the variables and regression coefficients with the factors; additionally, none of the variables were higher than 2.8 according to the standardized residuals (24,25). Several covariances have been observed between the variables as most of the GFIs were also within the acceptance limits. The confirmatory factor analysis was reapplied to the model of the scale, and results showed that the measurement model better matched the data after covariances (Table 3). In this context, the three-factor model of MGS is in accordance with the sample group and will be used without any change in the model of the scale and the variables were subdivided into factors similar to those of the original scale model according to the exploratory factor analysis.

### *Criterion-related validity*

For the criterion-related validity, the Turkish Patient Assessment of Choronic Illness Care was applied to the research group, and the correlation between the two scales was examined. According to the correlation coefficient (Pearson correlation) value, statistically significant positive correlations existed between the Turkish Patient Assessment of Choronic Illness Care and MGS_2_ of both adolescents (r=0.60, p<0.01) and parents (r=0.51, p<0.01).

### Reliability of MGS

### *Internal consistency*

For the Cronbach’s alpha internal consistency reliability coefficient, the values for MGS_1_ and MGS_2_ were 0.89 and 0.87 (adolescent) and 0.92 and 0.90 (parent), respectively. Table 4 lists the item-total score correlations and Cronbach’s alpha internal consistency coefficient values of the adolescent and parent scales and their sub-dimensions (management of the environment, provider characteristics, and process isues). The Cronbach’s alpha coefficient of the sub-dimensions of adolescent and parental forms ranged between 0.70-0.89 and 0.80-0.92 respectively.

### *Reliability of the scale*

The adolescent and parent MGS forms were reapplied to 44 adolescents and 56 parents, respectively, three weeks after the first implementation. The correlation coefficients (Pearson correlation) between the scale scores obtained in the two implementations were calculated. The test–retest correlation coefficients for adolescent and parent scales were 0.88 and 0.85, respectively (p<0.05). For the test–retest correlation coefficients, the values ranged between 0.45-0.89 for adolescent MGS_1_ and 0.51-0.84 for MGS_2_ (p<0.01); for the parents, the values were between 0.36-0.90 for MGS_1_ and 0.56-0.90 for MGS_2_.

## DISCUSSION

The MGS is a simple self-assessment scale designed to assess the health care satisfaction of adolescents with chronic conditions and their parents (8). In this study, the psychometric properties of the MGS in the Turkish sample were evaluated.

First, the scale was translated and back-translated from the original language into the target language to evaluate the language equivalence of the scale (26,27). Then, the scale items were examined by experts in terms of clarity and intelligibility for content validity. The scale assesses the individual care satisfaction in the transition period and was used in the adolescent and parent sample groups. The scale was considered as understandable and easy to apply.

The exploratory factor analysis was performed to examine the scarcely definable significant factors, which can be defined collectively by a large number of variables (26,27). The exploratory factor analysis of the adolescent scale resulted in a 22-item scale with 3 identified subscales that clarified 71% of the total variance, whereas that of the parent scale resulted in a 27-item scale with 3 identified subscales that clarified 73% of total variance (Table 1). The exploratory factor analysis results of Turkish MGS were similar to those of the original scale and proved the high structural validity of the Turkish MGS features. The variables were subdivided into factors similar to those of the original scale model according to the exploratory factor analysis (8). When we evaluated the factor loads of the items by principal components analysis and varimax orthogonal rotation technique, as expected, the item loads were higher than 0.30 (28).

The fitness of the model obtained by exploratory factor analysis was examined with GFI, and the results are shown in Table 2. The most commonly adopted ones are the resemblance rate (x^2^/df ), root mean square error of approximation, GFI, and adjusted GFI (29). Published reports indicated that values of x^2^/df ratio lower than 3.0 are considered as indicator of good fit, and those between 0 and 1 for root mean square residual and below 0.05 for root mean square error of approximation are desirable (23,24,26,29). Our study showed the good fit indicated by the x^2^/df ratio (2.49) and GFI and adjusted GFI. Although the GFIs were within acceptable fit limits, the other indices (comparative fit index, incremental fit index, Tucker–Lewis index, root mean square residual, and root mean square error of approximation) were beyond the acceptable ranges. Therefore, the model of the scale was analyzed in terms of modification indices and residuals, and causal relationships between the data and model fit indices were evaluated (25). None of the variables were excluded from the model given the high modification indices and regression coefficients of the factors; similarly, none of the variables were higher than 2.8 according to the standardized residuals (23,24,25). According to the results of the exploratory factor analysis, The item loads were not under 0.4, and the variables were subdivided into factors similar to the original scale model. Certain covariances have been observed between the variables as most of the GFIs were also within the acceptance limits. The confirmatory factor analysis was reapplied to the model of the scale, and the results revealed that the measurement model better matched the data after determining the covariances (Table 3). The fit indices obtained in our study support the acceptability of the structural model of Turkish MGS.

The Turkish Patient Assessment of Choronic Illness Care, which was developed with the same population and tested for validity and reliability, was performed to test the criterion validity. The correlation between the results of both scales was analyzed, showing a statistically significant relationship (positively, at a level of 0.01) between the total scores of Turkish Patient Assessment of Choronic Illnes Care and MGS_2_ scores of both adolescent and parent total scores (Adolescents: r=0.60, p<0.01; Parents: r=0.51, p<0.01). Both scales showed satisfaction with the current care. In our study, the results showed that MGS_2_ accurately assesses the current care satisfaction of the adolescents with diabetes and their parents in the period of transition.

The reliability of the scale was assessed by internal consistency using Cronbach’s alpha and item-total correlations. The item-total score correlation coefficient should be higher than or equal to 0.30, and the items with a value lower than 0.30 should be excluded (24,27). High correlation coefficiency values indicate the strong association of the scale items with the scale construct. The item-total score correlations of MGS_1_, MGS_2_, and their subscales were similar to those of the original scale and ranged between 0.36 and 0.83 (Table 4). In this context, a strong correlation exists between the items and the whole scale. Table 4 shows the Cronbach’s alpha internal consistency reliability coefficient values of the whole scale and sub-dimensions (management of environment, provider characteristics, and process issues). The Cronbach’s alpha values for the MGS_1_ and MGS_2_ totaled 0.89 and 0.87 (adolescents) and 0.92 and 0.90 (parents), respectively. The internal consistency of each sub-dimension was indicated by the Cronbach’s alpha values ranging between 0.71 and 0.92. High Cronbach’s alpha coefficients ​​indicate that the scale comprises consistent and balanced substances (17,22,24,26). The Cronbach’s alpha of the original entire scale for adolescents and parents were 0.91 and 0.94, respectively. Based on these results, our study obtained alpha coefficient values similar to the findings of Shaw et al. (8).

In conclusion, the “MGS” adapted to Turkish is a valid and reliable tool to assess the satisfaction and determine the health care expectations and needs of Turkish adolescents with diabetes and their parents.

## Figures and Tables

**Table 1:**
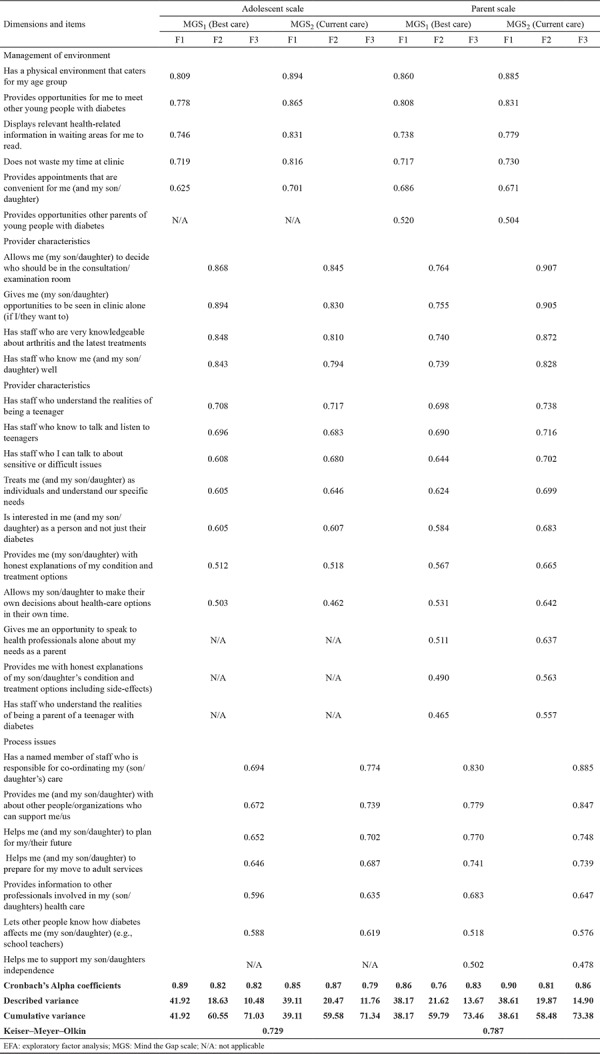
EFA results of adolescent and parent MGS_1_ and MGS_2_

**Table 2:**
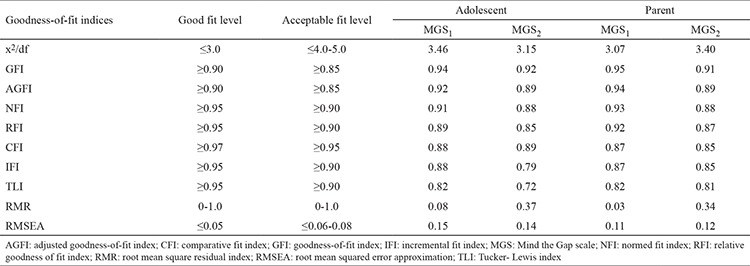
Goodness-of-fit indices of the model

**Table 3:**
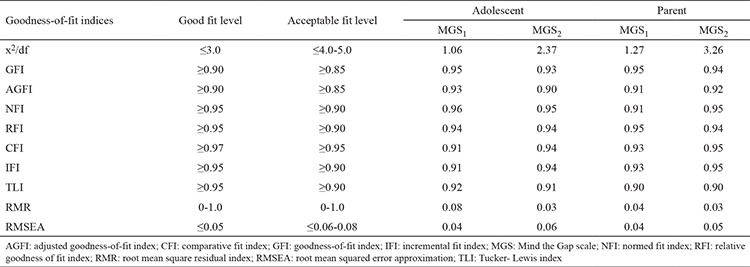
Goddness-of-fit indices of the model after covariances between the items

**Table 4:**
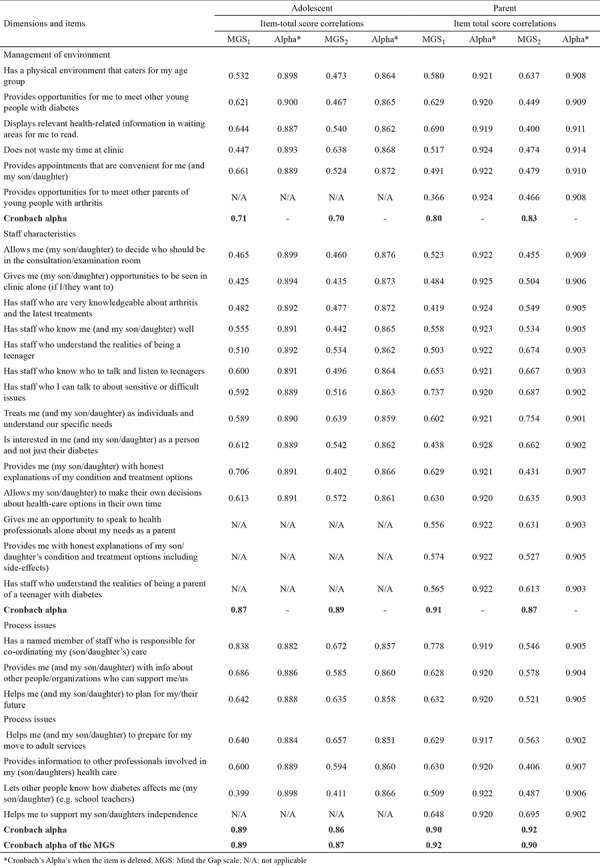
Item-total score correlations and Cronbach’s Alpha of MGS
